# A Chinese case of fragile X-associated tremor/ataxia syndrome (FXTAS) with orthostatic tremor:case report and literature review on tremor in FXTAS

**DOI:** 10.1186/s12883-020-01726-z

**Published:** 2020-04-20

**Authors:** Cuiping Zhao, Yiming Liu, Yihua Wang, Hongyan Li, Bin Zhang, Yaoxian Yue, Jianyuan Zhang

**Affiliations:** 1 Department of Neurology, Qilu Hospital, Shandong University, 44 Wenhua west Rd, Jinan, 250012 China; 2 Department of Neurosurgery, Qilu Hospital, Shandong University, 44 Wenhua west Rd, Jinan, 250012 China

**Keywords:** Fragile X-associated tremor/ Ataxia syndrome, Orthostatic tremor, *FMR1* gene,tremor, Ataxia

## Abstract

**Background:**

Fragile X-associated tremor/ataxia syndrome (FXTAS) is a late onset, X-linked genetic, neurodegenerative disorder caused by a “premutation (PM)” in the fragile X mental retardation 1 (*FMR1*) gene. Here we report a case of FXTAS from mainland of China who presented with rare orthostatic tremor. A review of tremor of FXTAS in the literature is also included.

**Case presentation:**

A 67-year-old right-handed farmer started with tremor of both legs 8 years ago which was present while standing but absent when sitting or lying and progressed with unsteady gait one and a half years ago. The brain MRI showed high intensity signal in the bilateral middle cerebellar peduncles (MCP) in T2-weighted and fluid-attenuated inversion recovery (FLAIR) images and gene test for premutation for *FMR1* was positive with 101 CGG repeats. The patient met the the diagnosis of definite FXTAS. Clonazepam and topiramate were administered to control tremor. We reviewed the literature and identified 64 cases with detailed clinical and genetic information. Orthostatic tremor associated with FXTAS is very rare. We found 85.2% patients reported tremor,42.6% with intention tremor,36.1% with kinetic tremor,32.8% with rest tremor and 29.5% with posture tremor. 37.7% of patients who have tremor showed at least two types of tremor. There were 6 patients with isolated rest tremor. There was 2 patient with voice tremor and 6 with head tremor. We also found that 74.6% FXTAS patients had family history of *FMR1* gene associated diseases including Fragile X syndrome (FXS), FXTAS or fragile X-associated primary ovarian insufficiency (FXPOI).

**Conclusions:**

Adding our data to the available literature suggests that orthostatic tremor could be a rare initial manifestation of FXTAS and the review will increasing our understanding the phenotype of tremor in FXTAS. Family history of *FMR1* gene associated diseases might be an important clue to the diagnosis.

## Background

Fragile X-associated tremor/ataxia syndrome (FXTAS) is a late onset X-linked genetic neurodegenerative disorder caused by a “premutation (PM)” 55–200 CGG repeat expansion in the fragile X mental retardation 1 (*FMR1*) gene. The normal number of CGG of *FMR1* is less than 45 CGG repeats in the 5′ UTR region,gray zone contains 46–54 repeats (carriers with either movement disorders or memory complaints), premutation contains 55–200 repeats (causes FXTAS or FXPOI) and full mutation > 200(causes Fragile X syndrome). FXTAS mostly affects middle-aged and elderly men of 50–70 years old. The main motor features include tremor and cerebellar ataxia but there is high phenotypic variability with some carriers demonstrating parkinsonism, peripheral neuropathy, executive function deficits, dementia, and neuropsychiatric problems. The syndrome can mimic many common neurodegenerative disorders such as Parkinson’s disease (PD),multiple system atrophy (MSA), Alzheimer disorders (AD), essential tremor (ET), and pure ataxia. Tremor is seen in 48–80% patients and variable in different studies and intention tremor is the most common pattern [1, 2]. But it’s very rare that FXTAS patients present with orthostatic tremor (OT). Here we report an old man with FXTAS from mainland of China who had OT as initial manifestation for 8 years. We also review more than 64 cases in the literature to find out the spectrum of tremor and other phenotype.

## Case presentation

**Fig. 1:**
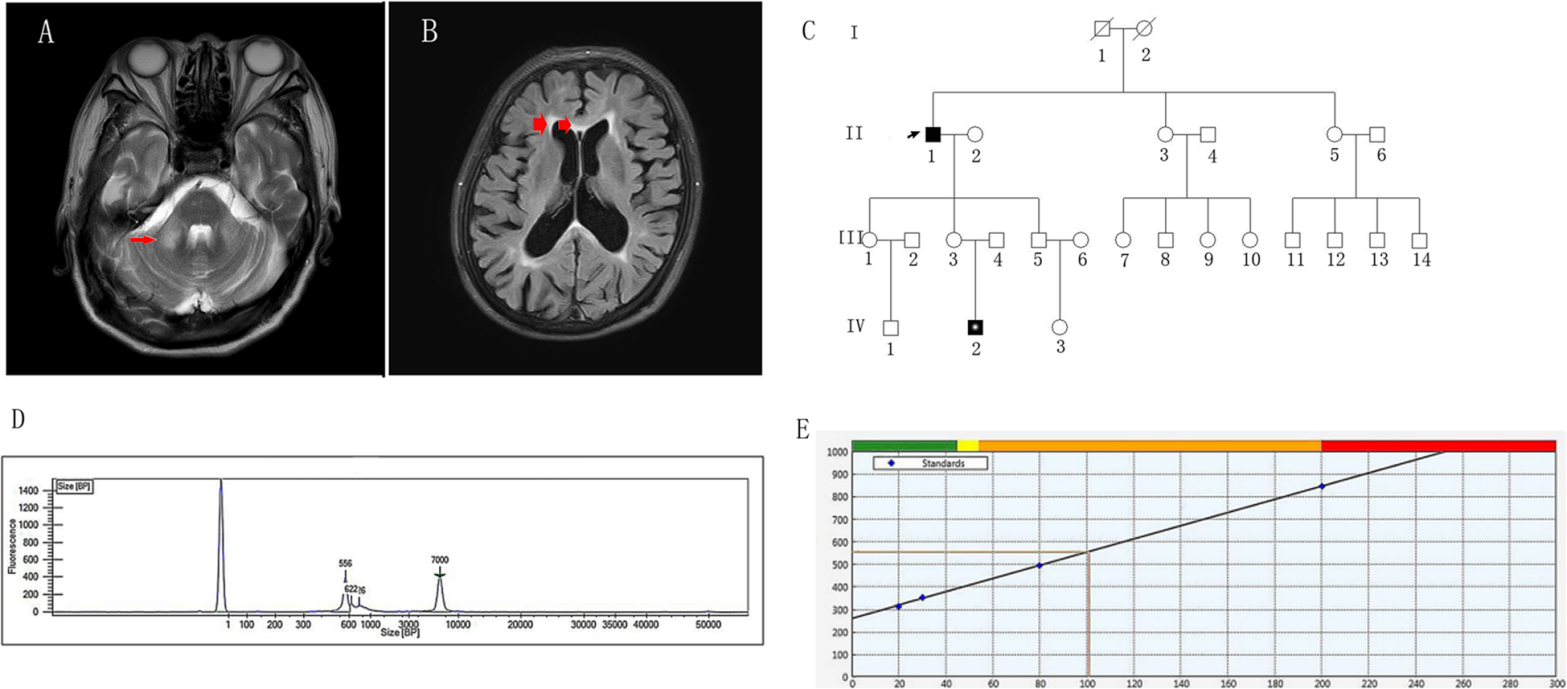
**a**: Brain MRI of the patient showed symmetric T2 hyperintensity in bilateral medipeduncle (MCP sign,red arrow); **b**:showed abnormal FLAIR intensity in periventricular white matter area and corpus callosum (red arrow); **c**:Pedigree of the case. II1 was the patient with FXTAS. III 3 was his younger daughter who stopped the menstruation at her 30s.IV2 was the son of his youngest daughter and had autism and attention deficit hyperactivity disorder (ADHD). But III 3 and IV2 denied the gene test; **d**: Results of CGG repeat PCR of FMR1. The X⁃coordinate represents the fragment size and the Y⁃coordinate is the peak height. The fragment size of this patient is 556 bp, corresponding peak height is 396 bp, and other peaks are the reference materials for detection. **e**: Linear regression graph between CGG repeat numbers and main peak length. The intersection of yellow lines indicates the fragment size of this patient is 556 bp and the number of CGG repeats is 101

A 67-year-old right-handed farmer from the mainland of China was admitted to neurology department with slowly progressive tremor in limbs for 8 years. The tremor started from both lower limbs. It was only present while standing but absent when sitting,lying or walking. He had no problem in initiating the gait and no fall. The patient was diagnosed with essential tremor for long time and he was still functional in daily life without any medical therapy. Tremor became worse one and a half year ago and he felt unsteady. Tremor started in both arms 8 months ago which was remarkable when working with hands. Family history showed that his younger daughter stopped the menstruation at her 30s and the son of his youngest daughter had autism and attention deficit hyperactivity disorder (ADHD) (Fig. 1 C). Neurological examination revealed remarkable and visible tremor in both legs when standing still, intention tremor in both hands, mild postural tremor in both arms and rest tremor in left hand. He swayed on Romberg’s test and had difficulty with tandem gait. He hadn’t finger-nose and heel-shin incoordination,rigidity of limbs or nystagmus in eyes. The finger tapping was slow and clumsy bilaterally. His cognitive evaluations with Mini-mental State Examination (MMSE) and Montreal Cognitive Assessment (MoCA) were 28/30(education state: primary school) and 20/30 respectively. There was no muscle weakness, sensory disturbance or orthostatic hypotension. Laboratory tests including blood cell counts, liver function, kidney function,thyroid function, ceruloplasmin and homocysteine were within the normal range. We found no abnormalities in the cerebrospinal fluid. Nerve conduction study values were within the normal range. The T2-weighted and fluid-attenuated inversion recovery (FLAIR) brain MRI indicated high intensity signals in the bilateral middle cerebellar peduncles (MCP),multiple sporadic high intensity signals in the cerebral white matter and atrophy of the cerebral cortex and cerebellum (Fig. 1a,b). FXTAS were considered and test for permutation of *FMR1* gene was performed and showed positive with 101 CGG repeats (Fig. 1d,e). The patient met the the diagnosis criteria [3] with definite FXTAS. Because the heart beat was very low (50–60/min) β-receptor blocker was not used. Clonazepam (0.5 mg–1 mg qn) and topiramate (primidone was not available) were administered and the tremor was relieved a little.

## Review on tremor in FXTAS

We conducted a systematic review of the literature to identify primary clinical case report and case studies reporting individuals who were FXTAS diagnosed with clinical manifestation and gene testing. We searched for all English language papers published between January 2001 and June 2019 in PubMed with the term (“Fragile X-associated Tremor Ataxia Syndrome” OR “FXTAS” and limited to case reports of patients who were (1) positive for premutation for the *FMR1* gene and (2) described the clinical manifestations including tremor,ataxia,cognitive condition, parkinsonism and other symptoms in details especially about tremor. We analyzed and summarized the characters of these cases.

**Fig. 2:**
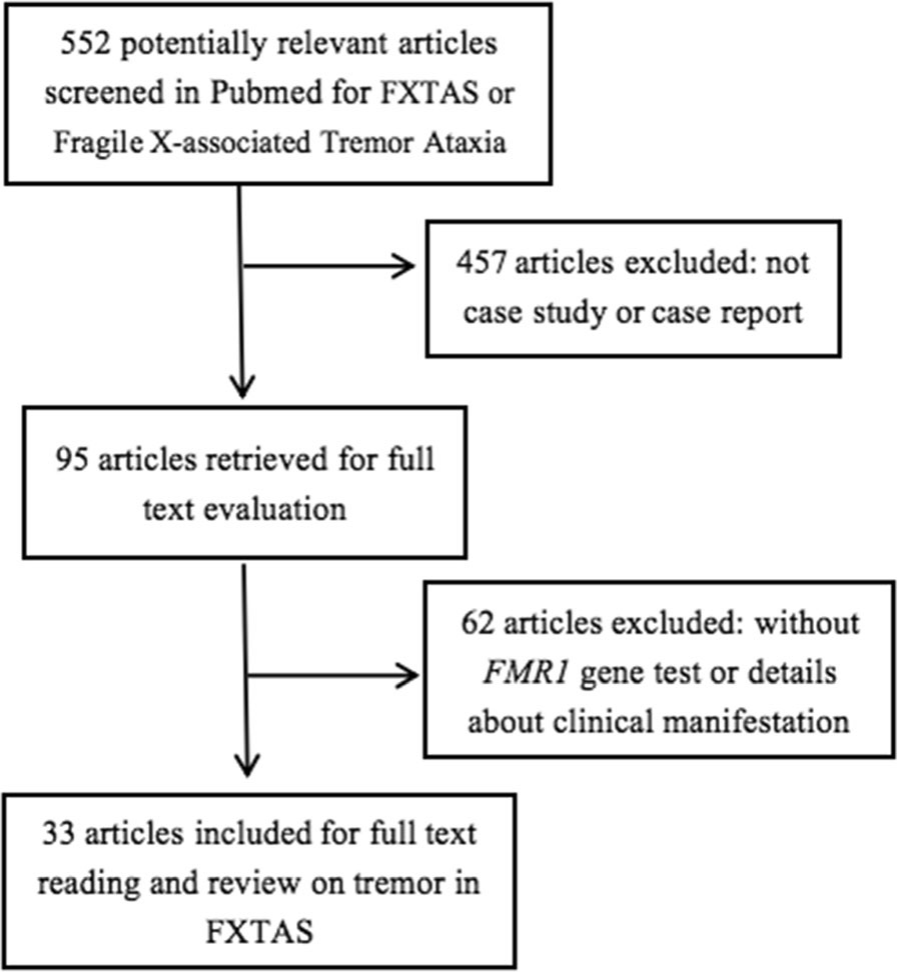
Inclusion and exclusion process of relevant literature for FXTAS case study or report in which the patients were diagnosed with clinical manifestation and *FMR1* gene testing with detailed description of the clinical manifestation especially tremor

In total, we searched 552 articles with term Fragile X-associated Tremor Ataxia Syndrome, we got 95 articles when limited to case report. We identified 33 articles reporting 64 patients fulfilling our inclusion criteria (Fig. 2). Reference 4 reported 19 patients with clinical information summarized in Tables but only 4 cases described in details so we included these 4 cases. The main findings of the review are summarized in Table 1 . We analyzed the clinical character and outlined in Table 2.

**Table 1:** Clinical features of patients with FXTAS in the literature

Case	Sex	Onset age (y)	Duration (y)	Primary diagnosis	Initial symptom	Kinetic Tremor	Intention tremor	Postural tremor	Rest tremor	Orthostatic tremor	Head or voice tremor	Ataxia	Cognitive problem	Parkinsonism	CGG repeat	Family history	Ref
1	M	63	8	ET	tremor	Y	N	Y	N	N	N	Y	Y	N	83	PD, FXS, ADHD	[1]
2	F	60	6	NA	gait	N	Y	N	N	N	N	Y	Y	N	77/77	neurological problem	[5]
3	M	52	3	NA	tremor	N	Y	N	N	N	N	N	Y	N	77	FXTAS	[5]
4	F	80	12	NA	gait	N	Y	N	N	N	N	Y	Y	N	30/77	FXTAS	[5]
5	M	58	8	ET	tremor	Y	Y	Y	N	N	N	Y	Y	N	95	FXPOI, mental retardation	[6]
6	M	54	11	NA	tremor	N	Y	Y	N	N	N	Y	Y	Y	98	FXS,PD	[7]
7	F	68	8	ET	tremor	N	N	Y	N	N	voice	Y	Y	N	31/95–105	FXS	[8]
8	M	55	4	Cerebellar atrophy	gait ataxia	N	N	N	N	N	N	Y	Y	Y	110	gait problem, parkinsonism,cognitive	[9]
9	F	54	4	MSA	gait ataxia	N	N	N	Y	N	N	Y	N	Y	29/135	N	[9]
10	M	54	11	NA	cognitive	Y	N	N	Y	N	N	Y	Y	Y	297–480	Fragile X family	[10]
11	F	41	26	NA	tremor	Y	Y	Y	Y	N	head	Y	N	Y	18/90	N	[11]
12	F	30	27	NA	tremor	N	Y	N	N	N	N	Y	N	N	29/93	FXS	[11]
13	F	75	10	NA	anxiety depression	N	Y	N	N	N	N	Y	N	N	29/87	FXS	[11]
14	F	52	10	NA	tremor	N	Y	N	Y	N	N	N	N	N	18/90	FXS	[11]
15	F	71	4	NA	Gait, tremor	N	Y	N	N	N	N	Y	N	Y	30/78	N	[11]
16	M	60	0.5	NA	tremor, gait	NA	NA	NA	NA	NA	head	Y	N	N	NA	NA	[12]
17	M	65	9	NA	tremor	NA	NA	NA	NA	NA	NA	Y	N	N	95	N	[12]
18	F	56	6	NA	gait	Y	Y	N	N	N	N	Y	Y	N	75	FXS	[13]
19	M	in late 20s	about 5	NA	tremor	N	Y	Y	N	N	N	Y	Y	N	88	FXTAS	[14]
20	M	58	2	NA	gait	Y	N	N	N	N	N	Y	Y	N	114	FXPOI	[15]
21	M	53	4	cerebellar ataxia	ataxia	N	N	N	N	N	N	Y	Y	N	100	balance disorder, FXPOI	[16]
22	M	65	2	NA	gait	N	N	N	N	N	N	Y	Y	Y	87	N	[16]
23	M	68	8	NA	Gait ataxia	N	N	N	Y	N	N	Y	Y	Y	78	FXS, FXPOI	[17]
24	F	About 65	About 5	NA	gait tremor	N	N	Y	N	N	head	Y	Y	N	95	FXS	[18]
25	M	about 60	About 5	NA	tremor	N	Y	N	Y	N	N	Y	Y	N	75	FXS	[19]
26	M	62	0	NA	cognitive	NA	NA	NA	NA	NA	NA	Y	Y	N	59–200	N	[20]
27	M	63	1.5	NA	personality change	N	N	N	N	N	N	Y	Y	Y	> 90	FXS	[20]
28	M	64	6	NA	ataxia	N	Y	Y	N	N	N	Y	Y	Y	mosaic 180–410,	FXS	[21]
29	M	61	4	NA	tremor	NA	NA	NA	NA	NA	NA	Y	Y	N	93	FXS.	[22]
30	M	44	14	NA	Autonomic Symptom	N	Y	N	N	N	N	Y	Y	N	20–800 mosaic	FXS	[23]
31	F	60	5	NA	cognitive	N	Y	Y	N	N	head	Y	Y	N	57	N	[24]
32	M	66	0	NA	tremor	N	Y	N	N	N	N	Y	Y	Y	200	N	[25]
33	M	49	14	CMT	numbness	N	Y	N	N	N	N	Y	Y	N	103	N	[26]
34	M	58	6	CMT	weakness, numbness	N	Y	N	N	N	N	Y	N	N	72	N	[26]
35	M	59	7	NA	gait ataxia	N	Y	N	N	N	N	Y	N	N	116	N	[26]
36	F	61	4	NA	numbness pain	N	Y	N	N	N	N	Y	N	N	20/79	N	[26]
37	M	73	0	NA	through a family research study	N	N	N	N	N	N	N	N	N	64	FXS	[26]
38	M	77	3	NA	diplopia	N	Y	N	Y	N	N	Y	Y	N	NA	FXS	[27]
39	M	45	1	PD	tremor	N	N	N	Y	N	N	N	Y	Y	60	N	[28]
40	F	76	10	PD	tremor	N	N	N	Y	N	N	N	Y	Y	60	with mutation of FMR1	[28]
41	F	68	5	PD	tremor micrographia	N	N	N	Y	N	N	Y	Y	Y	86	with mutation of FMR1	[28]
42	M	60	12	PD	balance problem	N	N	N	Y	N	head	N	N	Y	64	With mutation of FMR1	[28]
43	M	64	2	depression	pathological crying	N	N	N	N	N	N	N	Y	N	NA	N	[29]
44	M	56	7	ET	tremor	Y	N	Y	N	N	N	Y	N	N	155	tremor	[30]
45	M	50	4	PD	tremor	Y	N	Y	Y	N	N	Y	Y	N	109	N	[31]
46	F	56	6	NA	Sensation problem	N	N	N	N	N	N	Y	N	N	88	FXS	[32]
47	M	71	3	NA	gait	Y	Y	N	N	N	N	Y	Y	N	88	FXS	[33]
48	M	65	5	NA	cognitive	Y	N	N	N	N	N	Y	Y	N	104	FXS, FXTAS	[33]
49	M	65	3	NA	cognitive	N	N	N	N	N	N	N	Y	N	110	FXS, FXTAS	[33]
50	M	67	NA	NA	unsteadiness	Y	N	Y	N	N	N	N	N	N	57	FXS	[34]
51	M	46	7	NA	anxiety	N	N	N	N	N	N	N	N	N	56	FXS	[34]
52	M	61	2	NA	fall attack	N	Y	N	N	N	N	Y	N	N	99	FXS	[34]
53	M	67	3	NA	balance problem	Y	N	N	N	N	N	Y	Y	Y	99	FXS, FXPOI	[4]
54	F	74	1	NA	balance problem	Y	N	N	N	N	N	Y	Y	Y	90/29	FXS	[4]
55	F	65	10	Plantar fasciitis	gait	N	N	N	Y	N	N	Y	Y	Y	88	FXPOI, FXTAS	[4]
56	M	77	3	PD	gait	Y	N	N	Y	N	N	Y	Y	Y	68	FXS	[4]
57	F	56	3	NA	tremor	Y	Y	Y	N	Y	N	Y	Y	Y	82	FXS	[35]
58	M	72	9	NA	tremor	Y	NA	Y	Y	N	N	Y	Y	Y	85	FXS	[35]
59	M	50	22	NA	tremor	Y	Y	Y	Y	N	N	Y	Y	Y	90	with mutation of FMR1	[35]
60	M	65	9	NA	cognitive	Y	NA	Y	Y	N	Head,voice	Y	Y	Y	87	FXS	[35]
61	M	NA	NA	NA	NA	Y	NA	N	N	N	NA	Y	Y	Y	110	NA	[35]
62	M	NA	NA	NA	NA	Y	NA	Y	Y	N	NA	Y	Y	Y	71	NA	[35]
63	F	NA	NA	NA	NA	Y	NA	Y	Y	N	NA	Y	Y	Y	NA	NA	[35]
64	M	NA	NA	NA	NA	Y	NA	Y	Y	Y	NA	Y	Y	Y	100	NA	[35]

*M* Male, *F* Female, *PD* parkinson’s disease, *ET* essential tremor, *NA* not available, *CMT* Charcot-Marie-Tooth, *MSA* multiple system atrophy, *FXS* Fragile X syndrome, *FXPOI* fragile X-associated primary ovarian insufficienc,*Y* with the symptom,*N* without symptom, *ADHD* autism and attention deficit hyperactivity disorder, *FMR1* fragile X mental retardation 1, *FXTAS* Fragile X-associated tremor/ataxia syndrome

**Table 2:** Data summary of Clinical Characteristics of of reported FXTAS cases

	Total(***n*** = 64)	Male (***n*** = 44)	Female (***n*** = 20)	***P***-value^b^
**Onset age (years old)**	60.5 (12.5)^a^	59.0 (10.5)	61.5 (14)	0.376
**Duration before diagnosis (years)**	5 .5(5.4)^a^	4.8 (5.6)	5.8 (5.2)	0.026
**Positive family history(%)**	44/59(74.6%)	30/40(75.0)	14/19(73.7%)	0.905
**Tremor(%)**	52/61(85.2%)	33/41(80.5%)	19/20(95.0%)	0.135
**Intention tremor(%)**	26/61(42.6%)	15/41(36.6%)	11/20(55.5%)	0.171
**Kinetic tremor(%)**	22/61(36.1%)	17/41(41.5%)	5/20(25.0%)	0.209
**Rest tremor(%)**	20/61(32.8%)	13/41(31.7%)	7/20(35.0%)	0.802
**Isolated rest tremor**	6/61(9.8%)	2/41(4.9%)	4/20(20.0%)	0.063
**Posture tremor(%)**	18/61(29.5%)	12/41(29.3%)	6/20(33.3%)	0.952
**Orthostatic tremor**	2/61(3.3%)	1/41(2.4%)	1/20(5.0%)	0.603
**voice tremor**	2/61(3.3%)	1/41(2.4%)	1/20(5.0%)	0.603
**Head tremor**	6/61(9.8%)	3/41(7.3%)	3/20(15%)	0.346
**More than two types of tremor(%)**	23/61(37.7%)	17/41(41.5%)	6/20(30.0%)	0.386
**Ataxia (%)**	52/64(81.3%)	35/44(79.6%)	17/20(85.0%)	0.604
**Parkinsonism(%)**	28/64(43.8%)	19/44(43.2%)	9/20(45.0%)	0.623
**Cognitive impairement(%)**	46/64(71.9%)	34/44(77.3%)	12/20(60.0%)	0.154

^a^ Median (interquartile range)

^b^ Quantitative data is compared by Mann-Whitney U test and categorical data is compared by Fisher exact test between male and female patients

## Discussion and conclusion

Here we report a rare case of FXTAS that OT was as initial manifestation for a long time. Studies have showed tremor in approximately 77% of men with FXTAS [36]. The tremor in FXTAS is typically bilateral intentional, postural or kinetic tremor in upper limbs, and although rest tremor may be seen in some patients it is often accompanied by intention tremor. Apartis et al. [37] reported that total of 86% of patients had tremor,action tremor resembling the tremor of ET in 35% of the patients, cerebellar intention tremor and postural tremor in 29%, and unilateral upper limb rest tremor in 12% in a study of 17 FXTAS patients using tremor recordings from a neuropack device.

The orthostatic tremor, also known as “shaky legs syndrome” was first coined in 1984 by Heilman and is an intriguing and rare condition, characterized by unsteadiness and tremor when standing that is relieved when sitting or walking which primarily affects the legs and trunk. As there are no published population-based epidemiological data, the prevalence and incidence of OT are unknown. In the Neurological Disorders of Central Spain (NEDICES) study [38], one group detected one OT patient in a cohort of approximately 4000 elderly subjects (data not published) [39]. Only recently there was a study reported othostatic tremor in their FXTAS cohort [35]. There is a broad spectrum in differential diagnosis of symptomatic OT, including non-tumoral aqueduct stenosis,chronic relapsing polyradiculoneuropathy, pontine lesions (such as Cavernoma, Tuberculoma), spinocerebellar ataxia type 2, small cell lung cancer, stiff-person syndrome, Graves’ disease and etc. [39] but rarely considering FXTAS. The tremor in both legs in our patient was orthostatic tremor according to the definition [40]. Idiopathic OT manifests with a high-frequency tremor (13–18 Hz). Fast (high frequency) OT may not be visible on routine examination, sometimes be palpable as a fine- amplitude rippling of leg muscles and might be heard noise using a stethoscope on the muscles of the legs but the patients rarely report tremor sensation as a presenting symptom. Slow OT(< 13 Hz) is usually sensed and reported by patients and visible on examination. In our patient it looked like low frequency though we didn’t perform tremor anyalysis with tremorgram. There were other similar condition should be considered to differentiate from OT. Orthostatic myoclonus (OM) could be confused with OT, which also causes unsteadiness on standing and improves with walking or sitting but OM patients usually have non-rhythmic, synchronous and have difficulty in initiate gait. OM patients usually can’t stand for long time because of jerk movement and orthostatic intolerance. Making the distinction between OM and OT requires electrophysiological studies. Unlike OT, the bursts are shorter in duration, non-rhythmic, and irregular. Our patient more likely had OT than OM because of other clinical aspects including the long history of the tremor with function, no fall and without problem of initiating gait.

In our review we found out that 85.2% patients reported tremor,42.6% with intention tremor,36.1% with kinetic tremor,32.8% with rest tremor,29.5% with posture tremor. 37.7% of patients with tremor showed at least two types of tremor. It was interesting that there were 6 patients with isolated rest tremor which was different from previous study [36, 37, 41]. There were 2 patients with voice tremor and 6 with head tremor which hasn’t been addressed before. Orthostatic tremor in associated with FXTAS and our findings in the review will make us better understand the spectrum of tremors in FXTAS.

The premutation is also associated with fragile X-associated primary ovarian insufficiency (FXPOI) in female and full-mutation carriers with over 200 repeats is associated with the Fragile X Syndrome (FXS), which is characterized by childhood-onset intellectual disability, seizures and autism. In western countries the *FMR1* premutation occurs in 1/800 males and 1/250 females, with FXTAS affecting 40–45% of male and 8–16% of female premutation carriers over the age of 50 [42]. It is estimated that there are many FXTAS patients in China because of the huge baseline population. From our review of the literature FXTAS was always misdiagnosed with PD,ET,MSA and other types of cerebellar ataxia. That is similar with the conclusion described in previous reports [43]. In mainland of China there were some studies which tried to find out FXTAS patients in many movement disorder cohorts. But there was negative result in screening *FMR1* gene within premutaion range in 201 PD,36 ET, 68 sporadic spinocerebellar ataxia, 32 MSA patients and healthy control. But if we select subjects in the individuals with high risk we will find more FXTAS patients. In our review we found out that 74.6% (44/59) FXTAS patients had family history of FXS, FXTAS and/or FXPOI. If we do family investigation in FXS children and FXPOI females we will find more FXTAS patients or premutation carriers.

In summary, we demonstrated orthostatic tremor as a rare potential clinical feature of FXTAS. Our review about the tremor in FXTAS and presentaion with OT in our patient might expand the spectrum of tremor associated with FXTAS. Our study also highlight that family history of FXS, FXTAS and FXPOI can be an important clue to the diagnosis.

## Data Availability

All data generated or analysed during this study are included in this published article.

## Abbreviations

FXTAS: Fragile X-associated tremor/ataxia syndrome
*FMR1*: fragile X mental retardation 1 gene
PD: Parkinson’s disease
MSA: Multiple system atrophy
AD: Alzheimer disorders
ET: Essential tremor
OT: Orthostatic tremor
ADHD: Attention deficit hyperactivity disorder
MMSE: Mini-mental State Examination
MoCA: Montreal Cognitive Assessment
MCP: Middle cerebellar peduncles
OM: Orthostatic myoclonus
FXS: Fragile X syndrome
FXPOI: FXTAS and/or fragile X-associated primary ovarian insufficiency
